# A Resilience-Building App to Support the Mental Health of Health Care Workers in the COVID-19 Era: Design Process, Distribution, and Evaluation

**DOI:** 10.2196/26590

**Published:** 2021-05-05

**Authors:** Eddye A Golden, Micol Zweig, Matteo Danieletto, Kyle Landell, Girish Nadkarni, Erwin Bottinger, Lindsay Katz, Ricardo Somarriba, Vansh Sharma, Craig L Katz, Deborah B Marin, Jonathan DePierro, Dennis S Charney

**Affiliations:** 1 Hasso Plattner Institute for Digital Health at Mount Sinai Icahn School of Medicine at Mount Sinai New York, NY United States; 2 Digital Health Center Hasso Plattner Institute University of Potsdam Potsdam Germany; 3 Center for Stress, Resilience, and Personal Growth Icahn School of Medicine at Mount Sinai New York, NY United States; 4 Department of Psychiatry Icahn School of Medicine at Mount Sinai New York, NY United States; 5 Academic Informatics & Technology Icahn School of Medicine at Mount Sinai New York, NY United States; 6 Department of Pharmacology Icahn School of Medicine at Mount Sinai New York, NY United States; 7 Department of Neuroscience Icahn School of Medicine at Mount Sinai New York, NY United States

**Keywords:** mHealth, resilience, mental health, COVID-19, HCWs, digital health, health app, mental health platform, mobile phone

## Abstract

**Background:**

The COVID-19 pandemic has resulted in increased strain on health care systems and negative psychological effects on health care workers (HCWs). This is anticipated to result in long-term negative mental health effects on the population, with HCWs representing a particularly vulnerable group. The scope of the COVID-19 pandemic necessitates the development of a scalable mental health platform to provide services to large numbers of at-risk or affected individuals. The Mount Sinai Health System in New York City was at the epicenter of the pandemic in the United States.

**Objective:**

The Center for Stress, Resilience, and Personal Growth (CSRPG) was created to address the current and anticipated psychological impact of the pandemic on the HCWs in the health system. The mission of the Center is to support the resilience and mental health of employees through educational offerings, outreach, and clinical care. Our aim was to build a mobile app to support the newly founded Center in its mission.

**Methods:**

We built the app as a standalone digital platform that hosts a suite of tools that users can interact with on a daily basis. With consideration for the Center’s aims, we determined the overall vision, initiatives, and goals for the Wellness Hub app, followed by specific milestone tasks and deliverables for development. We defined the app’s primary features based on the mental health assessment and needs of HCWs. Feature definition was informed by the results of a resilience survey widely distributed to Mount Sinai HCWs and by the resources offered at CSRPG, including workshop content.

**Results:**

We launched our app over the course of two phases, the first phase being a “soft” launch and the second being a broader launch to all of Mount Sinai. Of the 231 HCWs who downloaded the app, 173 (74.9%) completed our baseline assessment of all mental health screeners in the app. Results from the baseline assessment show that more than half of the users demonstrate a need for support in at least one psychological area. As of 3 months after the Phase 2 launch, approximately 55% of users re-entered the app after their first opening to explore additional features, with an average of 4 app openings per person.

**Conclusions:**

To address the mental health needs of HCWs during the COVID-19 pandemic, the Wellness Hub app was built and deployed throughout the Mount Sinai Health System. To our knowledge, this is the first resilience app of its kind. The Wellness Hub app is a promising proof of concept, with room to grow, for those who wish to build a secure mobile health app to support their employees, communities, or others in managing and improving mental and physical well-being. It is a novel tool offering mental health support broadly.

## Introduction

COVID-19 has resulted in over 41 million infections with a worldwide case fatality ratio of approximately 2.6% as of early November 2020 [1,2]. Disease spread has been facilitated by a prolonged incubation period and variable symptomatology and severity. As a result, there is a tremendous burden on the health care system, with health care workers (HCWs) experiencing increased stress and demand and increased risk of COVID-19 infection, particularly for patient-facing staff [3]. With consideration for the existing issues of burnout and stress within the health care population, the additional impact of COVID-19 on HCWs, who represent a vulnerable group during this pandemic, is a growing concern [4-6]. Results from a survey of 2579 frontline HCWs at Mount Sinai Hospital in New York City during spring 2020 showed that 39% of respondents screened positive for COVID-19–related posttraumatic stress disorder (PTSD), generalized anxiety disorder, or depression. This finding displays the growing need for additional mental health support among HCWs as a result of the pandemic [7].

In recent years, mobile health (mHealth) apps on smartphones have become ubiquitous tools for personal health management and behavior tracking [8,9]. mHealth apps can provide individuals with continuous feedback on health status and progress, push notification reminders, and other useful engagement features. These remote, digital capabilities pose a major advantage for smartphones because they provide users with a mechanism to engage with, study, and interpret their health behaviors more regularly and comprehensively than in an average sequence of in-person doctors’ appointments or study visits [10,11]. Informed by the literature that has considered the techniques that drive behavior change through mHealth apps as well as their effectiveness [12-15], implementation of mHealth apps for personal health and behavior management can be fine-tuned and leveraged to become a powerful tool in behavior modification and overall lifestyle improvement.

mHealth apps have been rigorously implemented during the COVID-19 pandemic in order to support contact tracing, symptom tracking, and behavioral management for individuals across the globe. These apps have been deployed to support health care systems, governments, and communities to reduce the spread of COVID-19 and its negative long-term effects on the stress and well-being of individuals [16-18].

The growing knowledge and popularity around mHealth apps, in combination with the imperative to support frontline workers [7,19], allowed us to launch a mental health–focused smartphone app for HCWs in New York’s Mount Sinai Health System. It offers tools and support for users to bolster emotional well-being [20]. In its current state, the app is anonymous and the data collected in this phase will offer insight into how to optimize the features and content.

## Methods

### Overview

The Wellness Hub app is designed as a tool for general mental health maintenance and resilience and connects users to the mental health services available through the Center for Stress, Resilience, and Personal Growth (CSRPG) and Mount Sinai at large [21]. A team of psychiatrists and psychologists affiliated with CSRPG provided all the content for the app and partnered with the Digital Discovery Program at the Hasso Plattner Institute for Digital Health at Mount Sinai to lead the software development. With consideration for the Center’s aims, we determined the overall vision, initiatives, and goals for the Wellness Hub app, followed by specific milestone tasks and deliverables for development. Content and design framework were shared between the development team and CSRPG. The first version of the app was released after 2.5 months of iterative development.

The app is a standalone platform that hosts a suite of tools that users can interact with daily. While initially only available within Mount Sinai, we envisage the app being applicable and deployable in the future to other outside settings where there is a need to offer resilience-building tools to HCWs. Content (eg, lists of local supportive resources) could be tailored to specific health care systems.

### Resilience Content

Resilience-focused content and user tasks for the app were adapted from resilience training workshops provided to Mount Sinai Health System staff starting in July 2020, in direct response to the impact of the pandemic on staff well-being. The Center’s resilience workshops draw upon the work of Southwick and Charney, who outlined 10 factors contributing to personal resilience from laboratory research and interviews with trauma survivors, including prisoners of war during the Vietnam War [22].

The Center’s staff developed a peer co-led resilience training curriculum and first piloted a series of eleven 45-minute workshop meetings [21]. Virtual training of over 70 peer leaders was conducted over the summer of 2020, with an initial focus on nurses and physician assistants. As of November 2020, 46 unique staff members have participated in the workshops, with time constraints often cited as reasons for nonattendance. Based on these practical considerations, we condensed the intervention to five meetings in the fall of 2020 focusing on the following: (1) realistic optimism, (2) facing fears and active coping, (3) social support and utilizing resilient role models, (4) self-care, and (5) faith, meaning, and spirituality. Video content has been developed around these five topics hosted in the app—on Mount Sinai’s YouTube channel and on the Center’s website—to facilitate engagement of staff who do not have the time to participate in person [23].

### App Framework

#### Overview

We defined the app’s primary features based on the mental health assessment and needs of HCWs. Feature definition was informed by the results of a resilience survey widely distributed to Mount Sinai HCWs [7] and by the resources offered at CSRPG, including workshop content.

Most prominently, app users answer standardized mental health surveys with subsequent score-based feedback that incorporates various exercises for building resilience. Progress is visualized over time based on individual survey scores, showing green (good) or yellow (cautionary, screen-in) feedback depending on the score, allowing users to track their mental health in a regular, quantitative, and accessible way. A user will receive randomized feedback text based on their score (ie, good vs cautionary). If a user scores in a way that suggests a need for mental health care (ie, cautionary), a feedback screen appears suggesting that they seek additional mental health and wellness resources and lists available resources and tools directly on the screen for easy access. App users can write private digital journal entries and access videos for relaxation, including some created specifically for Mount Sinai employees. Users also have the ability to tailor their resources and videos to suit their mindset and interests by favoriting specific ones for ease of access. The app also offers quick links to Mount Sinai wellness-related resources (eg, spiritual care and behavioral health clinics). The app is intended to be used in conjunction with CSRPG’s resilience workshops and, therefore, includes key information and activities related to resilience development. We selected features based on their usability as it relates to mental health management and their compatibility with behavior change theory [12,24]. Our primary aim was to build features that support feedback and self-monitoring in a simple and straightforward manner through surveys and journaling and, in this way, support behavior change. Resources in the app—videos and referral resources—serve as reinforcement and added support if desired.

#### Mental Health Surveys

The app contains mental health surveys presented on the main screen. Users are first introduced to the surveys in a *baseline* fashion, where all questions are offered at one time, in one baseline survey. After completion, users will see each survey individualized on the home screen and can take each survey as many times as they would like. The surveys offered to users are validated measures commonly used in research and clinical practice, though they are available to answer consistently in the app as opposed to on a validated schedule. This is because the surveys are meant to serve as a feedback mechanism for users, where they can regularly assess their well-being and mental health as well as receive consistent feedback based on their results. The surveys were chosen based on prior knowledge about disorders that occur in the context of trauma as well as data derived from a previous survey distributed among Mount Sinai Hospital HCWs [7]. All surveys are presented to users in plain language, as opposed to their clinical name, with scores calculated as indicated in the original validated scoring instructions. The following surveys are included in the app:

1. Ecological mood assessment (“My Daily Feelings”). We included a daily feelings meter with emoticons indicating sadness to happiness on a scale from 1 to 7 for participants to rate their daily mood. This measure is similar to that utilized in prior research with smartphone apps [25].
2. PHQ-8 (8-item Patient Health Questionnaire) and GAD-2 (2-item Generalized Anxiety Disorder scale) (“My Mood”). The PHQ-8 and GAD-2 screen for depression and overall anxiety, respectively, over the past 2 weeks [26,27]. The PHQ-8 is identical to the PHQ-9, except that the item pertaining to suicidality is omitted [28]. PHQ-8 and GAD-2 items are presented on a scale from 0 to 3, with higher scores indicating more severe depression and anxiety, respectively. A PHQ-8 score of 10 or higher was used to indicate probable depression, while a score of 3 or higher on the GAD-2 suggested clinically significant anxiety problems.
3. PCL-5 (PTSD Checklist for DSM-5 [Diagnostic and Statistical Manual of Mental Disorders, Fifth Edition]) (“My Stress Reactions”). We utilized the PCL-5, a newly published brief 4-item version of the longer 20-item PCL-5, anchored to the COVID-19 experience, to evaluate posttraumatic stress reactions [29,30]. Items are presented on a scale from 0 (not at all) to 4 (extremely). Based on initial work with this brief measure, a score of 8 or higher was used as a cut point for probable PTSD [29].
4. CD-RISC2 (2-item Connor-Davidson Resilience Scale) (“My Coping Resources”). We utilized the CD-RISC2, a previously validated short form of the CD-RISC [31,32]. The scale ranges from 0 (not true at all) to 4 (true nearly all the time), with higher scores indicating greater resilience. Based on a review of the available normative data, a score of 5 or lower was used to indicate current problems with resilience for purposes of providing feedback [31].
5. WHO-5 (5-item World Health Organization Well-Being Index) (“My Well-being”). The WHO-5 is a 5-item globally utilized measure of overall well-being [33]. Items are presented on a scale from 0 (at no time) to 5 (all the time). Higher scores indicate greater well-being. Scores are summed and multiplied by 4 to yield an index ranging from 0 to 100; scores at or below 50 are suggestive of depression.
6. AUDIT-C (Alcohol Use Disorders Identification Test-Concise) (“My Alcohol Use”). The AUDIT-C is a widely utilized 3-item screening measure for alcohol misuse [34]. Items are presented on a scale from 0 to 4; scores of 3 or higher were taken to be suggestive of problems with alcohol misuse.
7. Spiritual struggle items (“My Spirituality”). Based on prior research, two items were included in the app to measure problems with spirituality based on prior research: (1) “Do you struggle with loss of meaning and joy in your life?” and (2) “Do you currently have what you would describe as spiritual or religious struggles?” [35]. These items were presented on a 4-point Likert scale ranging from 0 (not at all) to 3 (a great deal). Endorsement of 1 or higher on either item triggered feedback regarding addressing loss of meaning or spiritual problems.

**Figure 1:**
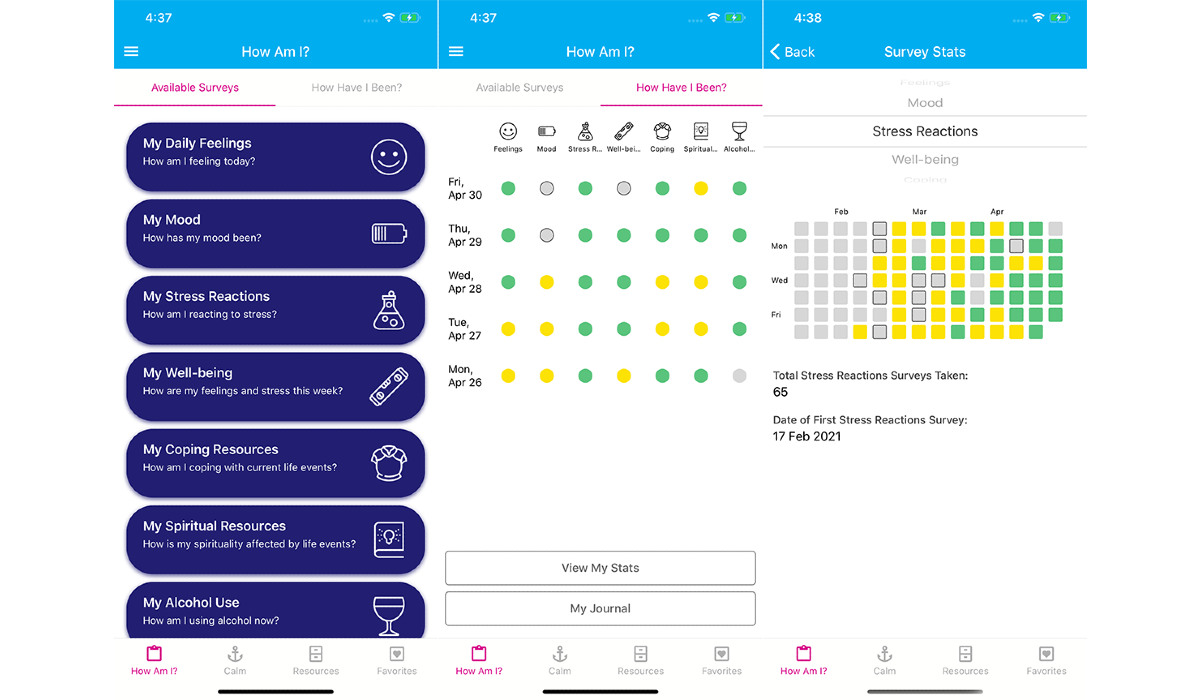
Available surveys (left), weekly progress screen (center), and survey-specific progress screen (right).

**Figure 2:**
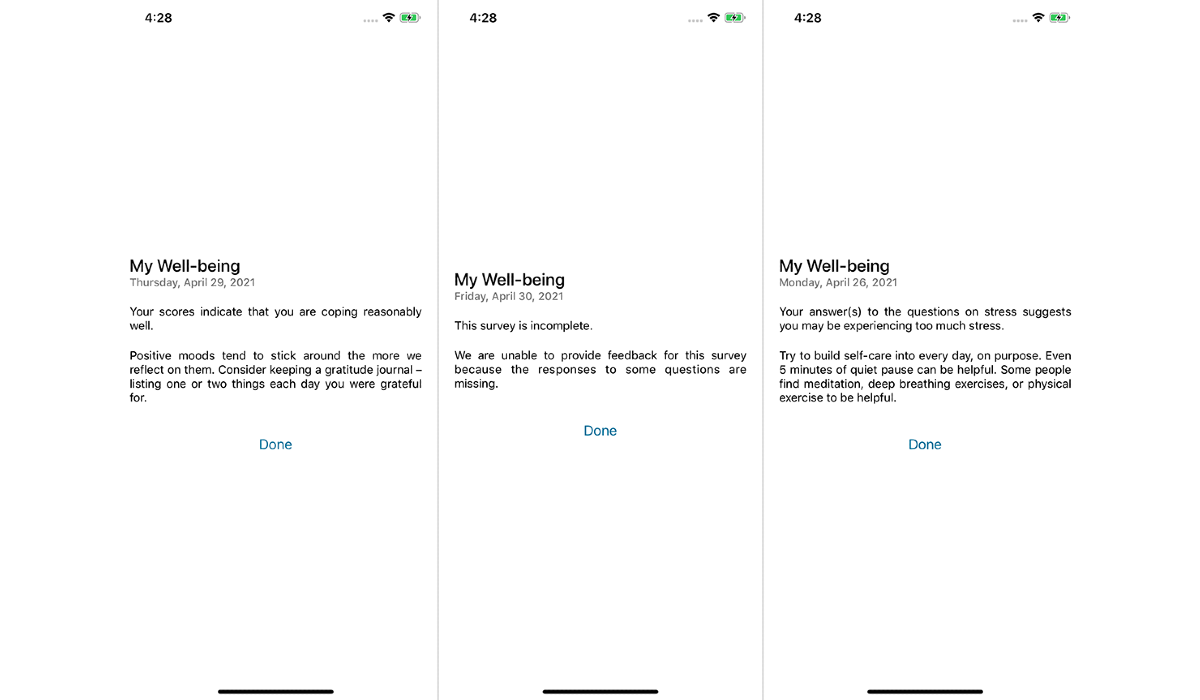
Randomized feedback based on survey score: good (left), incomplete (middle), or cautionary (right).

Figure 1 shows the available surveys on the app, as well as the weekly progress screen and the survey-specific progress screen. Figure 2 shows the randomized feedback sent to users based on their survey scores.

#### My Resources and My Journal

We offer a library of resources to our Wellness Hub users. Whether based on survey feedback, general curiosity, or additional mental health needs, the app provides access to the Mount Sinai mental health support network and offers resources and tools external to Mount Sinai.

For individuals looking for direct interaction with a mental health expert or support network, the app provides contact information for CSRPG, chaplaincy, and other behavioral practices and offices at Mount Sinai. We offer a page focused on promoting resilience and building a resilience plan. This page also offers additional CSRPG workshop content and videos.

**Figure 3:**
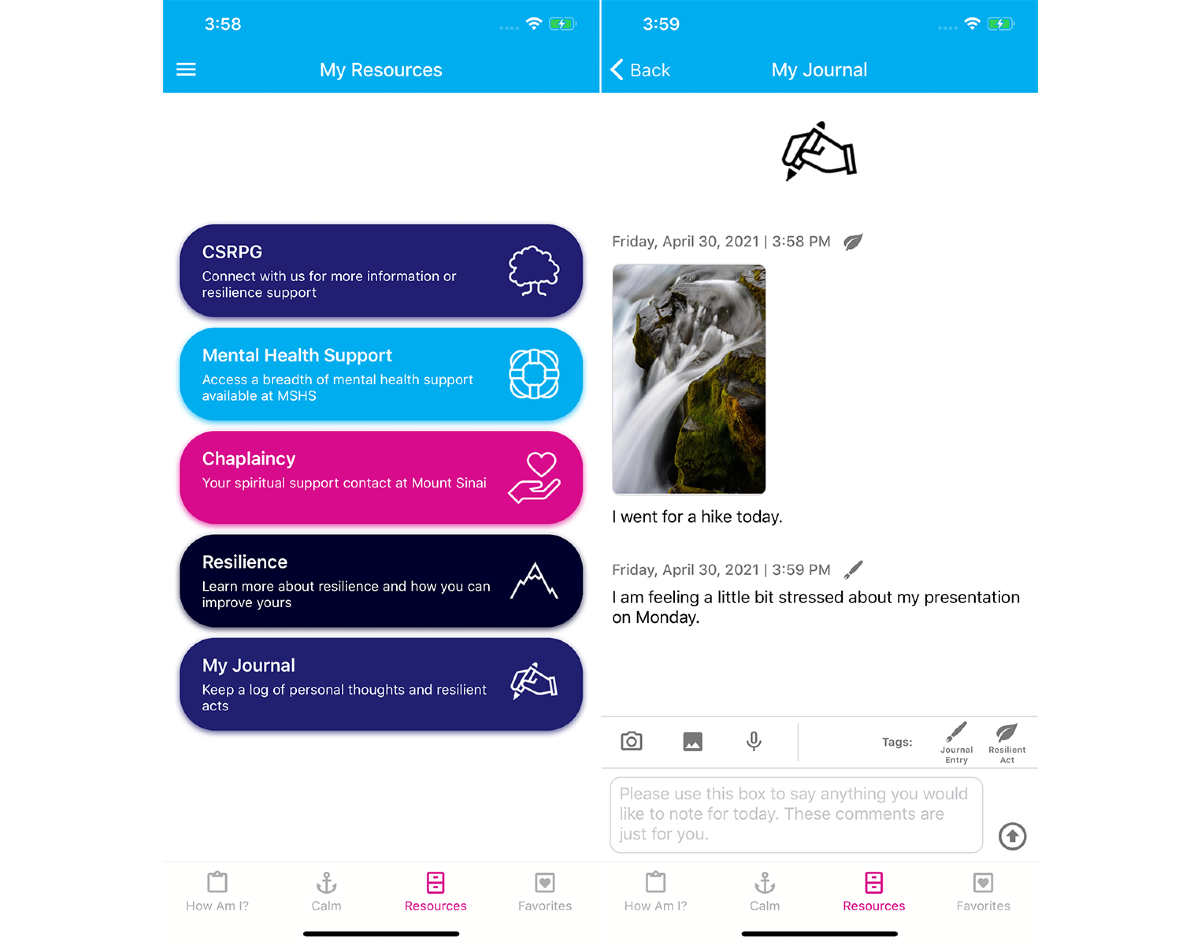
My Resources page, with My Journal feature showing tagged photo and text entries.

The “My Resources” tool includes a journal function, which allows users to write down any relevant thoughts or experiences that they feel are important to log. These entries serve as a way to self-monitor their feelings and thoughts and can be in the form of text, image, or voice recording. They can also be tagged as a regular journal entry or a resilience act. This tagging feature allows users to apply what they learn in the CSRPG workshops, along with the *Resilience* page in “My Resources” in order to discern for themselves what experiences are helping them to build their resilience (Figure 3).

Another feature provides HCWs with easily accessible relaxation content. The “My Calm” tool offers users a library of relaxation videos, some from the public web and some specifically designed for Mount Sinai employees [36]. These videos range in topic and duration so a user can select something that works for them in the moment. For example, an app user may choose a short, 2-minute video if they are on break during the workday, but may prefer to engage with a longer, more involved yoga video in the evening or to start the day.

#### General App Features

##### Overview

Wellness Hub also includes general app and health app–related features. Our data management page allows users to download their data and/or delete all data from the app. Because the app may hold sensitive or personal information related to a user’s feelings or mental health, we maintain an anonymous back-end server, meaning we do not collect identifiable data from any of our users. Our only way of identifying a specific user is if they provide us with their universally unique identifier that lives in the “My Profile” section of the app.

A notification-setting tool allows users to set the time of day they want to receive notifications about checking in with the app to engage with the available features. Users can also turn off notifications completely.

Additionally, and in accordance with standard practice, we also include information about the app, its developers, and additional copyright or reference information for content hosted in the app.

##### Data Privacy and Security

Data privacy and security are important considering the content of the app and its potentially stigmatizing and/or personal content, and we ensure that all anonymous data are stored with limited access within the Mount Sinai secure network. Further, all questions in the app are optional, including demographics.

Wellness Hub app data are hosted in a Health Insurance Portability and Accountability Act (HIPAA)-compliant ecosystem. For quality control, robust data transfer, and data persistence, the Wellness Hub app stores the data in an encrypted environment. The environment is fully encrypted, and the metadata are stored in secure non-Structured Query Language (NoSQL) instances for quality control.

**Figure 4:**
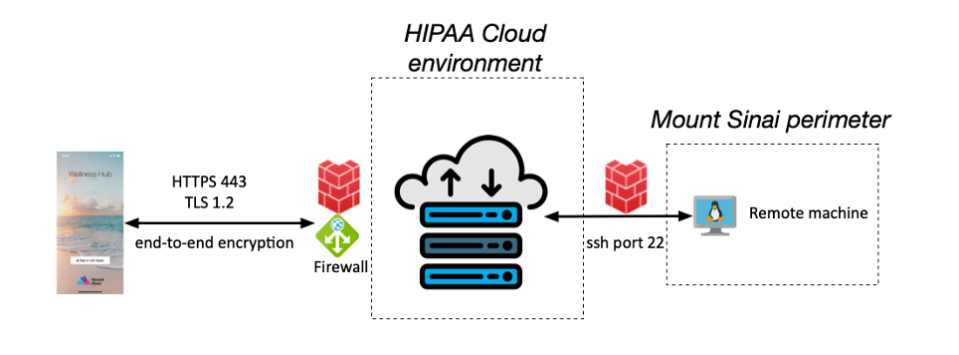
Data ingestion diagram. HIPAA: Health Insurance Portability and Accountability Act; ssh: secure shell; TLS: Transport Layer Security.

The data transferred from the app to the cloud are sent through an https protocol with end-to-end encryption. Authentication protection at the beginning of the data ingestion is performed, and all data requests external to the server are blocked. No personally identifiable information is ever transferred from the app to the back end. The server manager cannot identify the data’s owner, effectively making the ingested data anonymous (Figure 4).

The cloud system is only accessible through a secure shell with a public-private key, and IP address restrictions are in place to prevent cyberattacks. The database is restricted only to the app data and does not host any data from outside the app.

The app processes the survey scores locally and initializes the data transfer. Scores are given either a valid value or an invalid value, depending on whether the user completes a full survey or not. The app also implements single sign-on authentication, either Apple or Google, depending on which operating system the app is running. Consistent with our approach to privacy, the app has a nonutilization timer that expires if the app is not used for 2 minutes, and the timer forces a log-out when it expires.

##### Personalization

**Figure 5:**
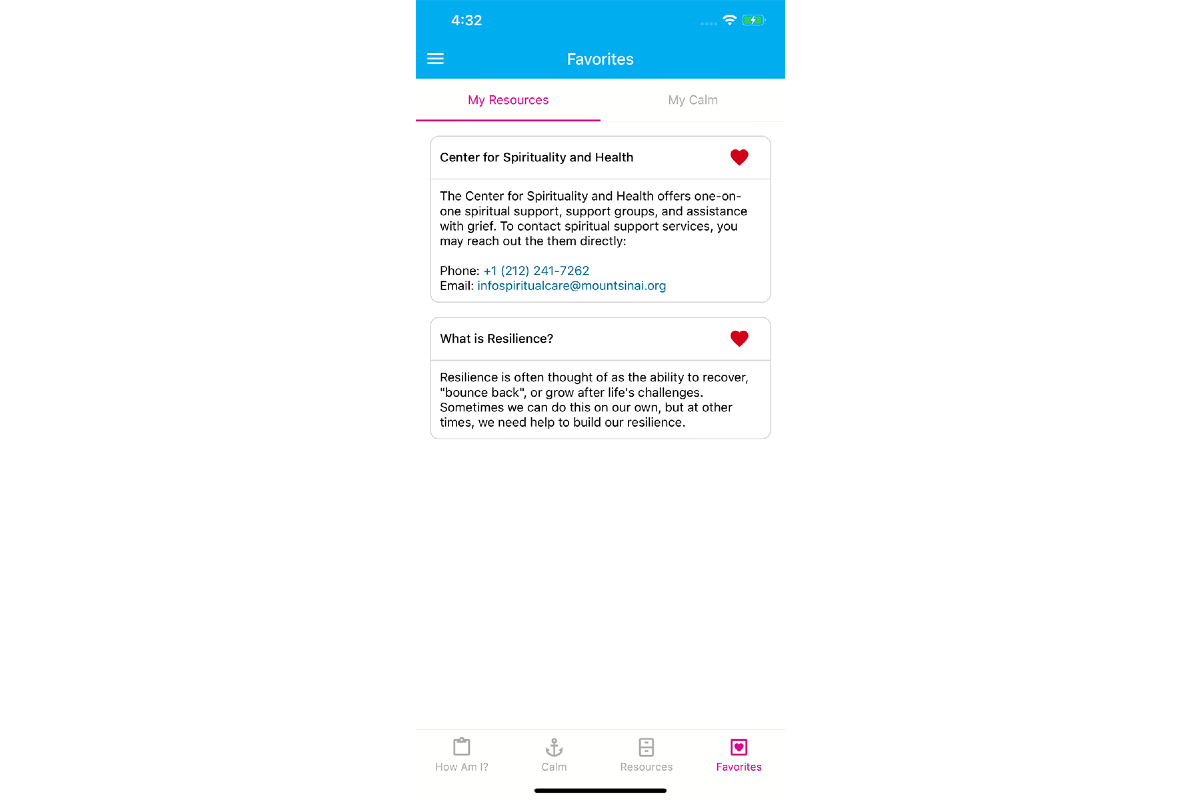
Example of the My Favorites page with resources and contact pages “hearted” and easily accessible.

The “Favorites” tool in Wellness Hub allows users to tailor the content of the app based on what they find most helpful to them. All resources and videos from the “My Resources” and “My Calm” pages can be selected as favorites and will subsequently appear as a personalized library in this view. Here, preferred resources can be easily accessible (Figure 5).

**Figure 6:**
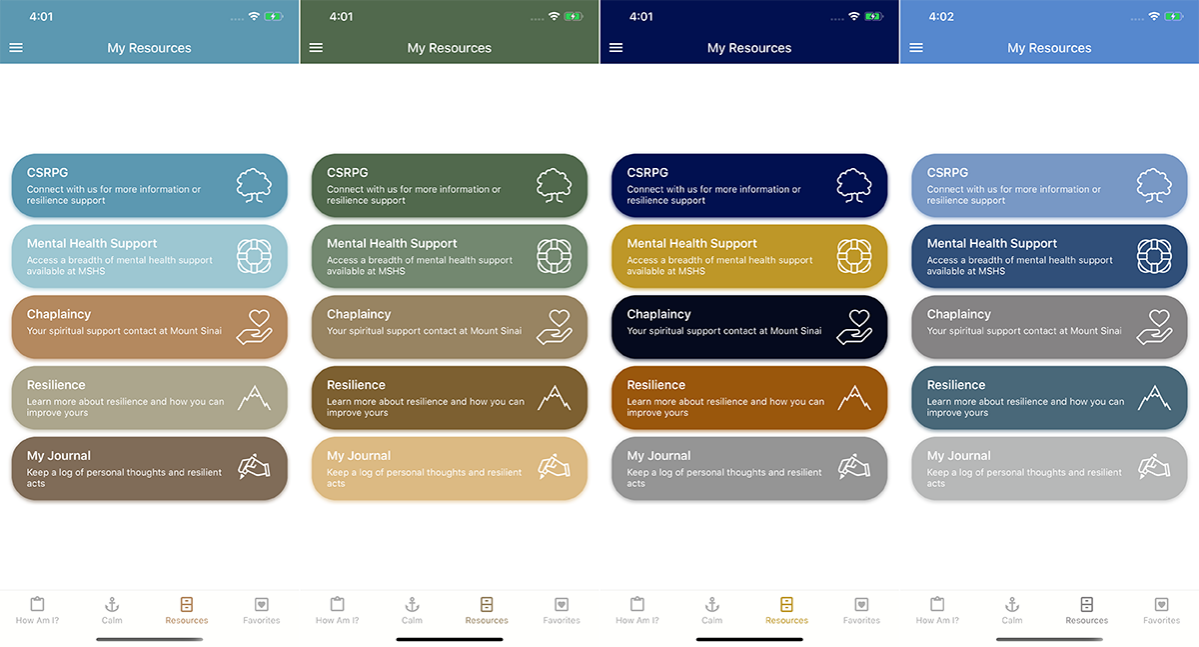
Tailoring the app by color scheme.

Beyond tailoring resources, we also allow users to further personalize their experience through design. In addition to the standard Mount Sinai colors, we include a feature allowing users to choose the color scheme they prefer for their app, with numerous options meant to offer a calming effect to the user (Figure 6). This serves as a simple way to allow personalization of the app, in the same way that they can personalize their favorite content and track their journal entries and survey responses.

##### Demographics

For future use, we have included a basic and optional “About Me” anonymous demographics survey in the standard profile section of the app in order to improve the personalization of resources based on age, role at Mount Sinai Health System, and other demographic or COVID-19–related features. This section can also help us better understand the breakdown of our user population and develop new or amend existing features to better address and target outreach to an underserved user population.

### Distribution

This app is available for both iOS and Android devices and is currently only available to employees of the Mount Sinai Health System. Due to this employment restriction in distribution, we developed this as a custom app that is only available for download using a unique redemption code or link. The code or link can only be used one time, thus limiting the possibility of users distributing the app outside of the health system. In order to verify the current employment status of the user, we created a page on the Mount Sinai intranet in which users must first log in, thus validating their employment, and subsequently select whether they want to download the app for iOS or Android. Next, they enter the email address to which they want to receive installation instructions. The intranet page automatically pulls a unique redemption code from a list on the back end and sends download instructions, including the unique code, to the interested employee. The code is then marked as “used” and unable to be supplied to another person.

The Wellness Hub app was launched between June and September 2020, as a Phase 1 pilot in which we manually distributed redemption codes to interested Mount Sinai Health System employees while further developing the app in anticipation of the Phase 2 launch in October 2020, to correspond with Mental Health Awareness Month. At the time of the Phase 2 launch in early October, CSRPG introduced the app to the Mount Sinai Health System community through regular presentations, in-hospital messaging, and email notifications.

### Key Performance Indicators

Prior to app development, we established key performance indicators (KPIs) to measure the success of the product and level of engagement of our app users. Our KPIs included total number of HCWs downloading the app, total number of baseline surveys submitted, number of users submitting additional surveys postbaseline, and general app engagement metrics (eg, number of app openings).

## Results

### Overview

This paper outlines the design and launch process for the Wellness Hub app. Development time for the platform was variable between Phase 1 and Phase 2. From initial ideation to the current version, we estimate a total of approximately 2.5 months for development and launch. This time frame is reflective of one software engineer, one product manager, and one designer working on the app. Since the app was developed in-house at Mount Sinai, the platform’s primary cost is for database management, which is a regular ongoing service fee. As of March 2021, there were new downloads each month and no reports of bugs or software crashes, suggesting a successful technical launch of the Wellness Hub app.

We analyzed several KPIs to determine the success of the app early after the launch. These early results serve to showcase the initial launch of the app. Between July 27 and October 13, 2020, during our Phase 1 pilot launch, which included manual app distribution and word-of-mouth recruitment, 74 users downloaded the app. We received informal feedback from many of these early users, which allowed us to refine the app and include additional features and adapt existing ones to support our user population. This led to the inclusion of relaxation videos and the improvement of our survey feedback content and progress visualizations.

Our broader, Phase 2 launch was on October 14, 2020, and included greater recruitment and easily accessible app distribution methods. Within 3 months of Phase 2, we received 157 additional app downloads, totaling our reach and app use to 231 users over the course of 4.5 months.

Of the 231 HCWs who downloaded the app, 173 (74.9%) completed our baseline assessment of all mental health screeners available in the app. As of 3 months after the Phase 2 launch, approximately 55% of users re-entered the app after their first opening to explore additional features, with an average of 4 app openings per person.

While the platform is meant primarily to provide individualized feedback to users, and not analyze a cohort of users together, we have compiled a brief overview of baseline results.

Slightly more than half of the baseline survey responses showed *screen-in* cautionary scores on the WHO-5 (97/173, 56.1%), AUDIT-C (79/159, 49.7%), and spiritual struggle items (113/159, 71.1%). This demonstrates a clear need for additional HCW support related to overall well-being and spiritual struggles (Table 1).

**Table 1:** Survey-specific completion at baseline and screen-in distribution.

Survey type	Total completed, n	Yellow score (screen-in), n (%)	Green score (doing okay), n (%)	Total incomplete, n (%)
Daily feelings	173	105 (60.7)	65 (37.6)	3 (1.7)
WHO-5^a^	173	97 (56.1)	71 (41.0)	5 (2.9)
AUDIT-C^b^	159	79 (49.7)	75 (47.2)	5 (3.1)
GAD-2^c^	102	38 (37.3)	64 (62.7)	0 (0)
PHQ-8^d^	160	45 (28.1)	105 (65.6)	10 (6.3)
PCL-5^e^	159	53 (33.3)	98 (61.6)	8 (5.0)
CD-RISC-2^f^	159	41 (25.8)	113 (71.1)	5 (3.1)
Spiritual struggle	159	113 (71.1)	40 (25.2)	6 (3.8)

^a^WHO-5: 5-item World Health Organization Well-Being Index.

^b^AUDIT-C: Alcohol Use Disorders Identification Test-Concise.

^c^GAD-2: 2-item Generalized Anxiety Disorder scale.

^d^PHQ-8: 8-item Patient Health Questionnaire.

^e^PCL-5: Posttraumatic Stress Disorder Checklist for DSM-5 (Diagnostic and Statistical Manual of Mental Disorders, Fifth Edition).

^f^CD-RISC-2: 2-item Connor-Davidson Resilience Scale.

On average, users took 2.06 (SD 6.5) surveys (screeners) after completing the baseline assessment. Of all the surveys available on the app, the daily feelings (“My Daily Feelings”), PHQ-8 (“My Mood”), and PCL-5 (“My Stress Reactions”) surveys had been taken the most, with 410, 368, and 318 total completions, respectively. Considering the everyday availability of these surveys for users on the platform, they were not taken on the validated schedule for which the surveys are primarily intended.

As our study of app usage proceeds, we will review resilience-related indicators beyond the baseline assessments taken by users. We intend to review additional KPIs that align with longer-term use of the platform.

### Strengths and Limitations of the App

The strength of the app is that the Center was able to quickly deploy a tool based on existing resilience literature in order to address a mental health need within the frontline HCW community amidst the initial wave of the COVID-19 pandemic.

The main limitation of the app is that, as it is not a research study in its current version, there is no strict protocol given to users for how they must interact with the app. As a result, there is significant variability in app usage and our ability to track user experiences relative to their demographics. Future versions of the app may include a research intervention that will empirically test the use and effectiveness of the app as a supplement to CSRPG workshops.

Additionally, we consider selection bias; users may already have a bias or inclination toward resilience building, or recognition of their need for wellness tools, thus leading them to search for an app-based tool. We may be missing an important HCW population that is both lacking in resources and would not know how to find or use this tool effectively. We have to consider an outreach strategy that will promote greater access or we must consider making these tools available in multiple formats and venues. Lastly, the app is available to a specific cohort with specific access: HCWs in the Mount Sinai Health System. In its current state, it is meant to serve partly as a supplement to CSRPG workshops held at Mount Sinai. This may impact external validation of the app’s efficacy in building resilience outside of this specific cohort.

## Discussion

mHealth apps are an increasingly used tool for people to maintain their physical and mental wellness [8]. mHealth apps focused on engaging features, linked to behavior change theory, are crucial in helping to empower people to take control of their own health [37].

There are many publicly available resilience-building platforms similar to Wellness Hub, such as COVID Coach and Calm. COVID Coach, developed by the US Department of Veterans Affairs, offers education about coping, self-care tools, progress visualization, and national mental health resources [38]. Calm is a meditation app that offers video and audio programs related to meditation and mindfulness [39]. The Wellness Hub app stands out due to its targeted nature, in that it provides specific and accessible health system resources to its users and also offers validated mental health surveys along with progress visualization. With HCWs feeling the brunt of the pandemic stress, it makes sense to offer them resources that are close to home and relevant to their work.

To address the mental health needs of HCWs during the COVID-19 pandemic, the Wellness Hub app was built and deployed throughout the Mount Sinai Health System. To our knowledge, this is the first resilience app targeted to HCWs and applied within the context of a global trauma. It includes validated surveys and questionnaires based on prior knowledge of disorders that occur in the context of trauma, a journal tool to allow users to log their feelings and activities related to resilience, and numerous Mount Sinai–specific resources and videos targeted to those seeking additional support and tools to build resilience. The app’s initial results, showing that more than half of the baseline assessments resulted in *screen-in* scores, are consistent with previous findings at Mount Sinai in the same cohort via a hospital-wide early pandemic survey [7].

The limited level of app use and engagement thus far may be related to the constrained recruitment strategies to date, considering the lack of in-person advertising, enrollment, and distribution due to the pandemic. Additionally, the slower uptake is consistent with our expectations of an initial app release, especially considering the involved download process and our active and occupied target audience of frontline HCWs and others in the health system.

We intend to continue developing this app by improving features based on feedback from our users, including the translation of the platform into Spanish and the integration of Mount Sinai employee and health care services, such as appointment scheduling. We will move beyond assessment by adding specific resilience-promoting interventions, action items, and tools. Potential future improvements include *badges* that users can obtain in order to encourage them through their resilience journey as well as inclusion of materials from the CSRPG workshop curriculum in specific *learning* sections and as thought prompts in an expanded version of our journal tool. As the CSRPG continues to grow and COVID-19’s effects continue to be felt across the health care system, we intend to use our KPIs, user feedback, and guidance from the Center workshops to determine the future direction of the app and its content.

The Wellness Hub app is a promising proof of concept for those who wish to build a secure mHealth app to support their employees, communities, or others in managing and improving mental and physical well-being. It offers a novel tool to offer mental health support broadly.

## Abbreviations

AUDIT-C: Alcohol Use Disorders Identification Test-Concise
CD-RISC2: 2-item Connor-Davidson Resilience Scale
CSRPG: Center for Stress, Resilience, and Personal Growth
DSM-5: Diagnostic and Statistical Manual of Mental Disorders, Fifth Edition
GAD-2: 2-item Generalized Anxiety Disorder scale
HCW: health care worker
HIPAA: Health Insurance Portability and Accountability Act
ISMMS: Icahn School of Medicine at Mount Sinai
KPI: key performance indicator
mHealth: mobile health
NoSQL: non-Structured Query Language
PCL-5: Posttraumatic Stress Disorder Checklist for DSM-5
PHQ-8: 8-item Patient Health Questionnaire
PTSD: posttraumatic stress disorder
WHO-5: 5-item World Health Organization Well-Being Index
